# Apolipoproteins and Lipoproteins in Health and Disease 2.0

**DOI:** 10.3390/ijms25116183

**Published:** 2024-06-04

**Authors:** Noemi Rotllan, Joan Carles Escolà-Gil

**Affiliations:** 1 Institut de Recerca Sant Pau, 08041 Barcelona, Spain; 2 CIBER de Diabetes y Enfermedades Metabólicas Asociadas (CIBERDEM), 28029 Madrid, Spain

Cardiovascular diseases (CVDs) remain the leading cause of mortality worldwide, accounting for 32% of global deaths, according to the World Health Organization (WHO). Traditionally, high- and low-density lipoprotein cholesterol (HDL-C and LDL-C, respectively) levels, along with their major protein components, apolipoprotein (apo) A1 and apoB, have been considered causal risk factors for CVDs. However, beyond CVDs, apolipoproteins and lipoproteins also play pivotal roles in other conditions such as metabolic syndrome, obesity, type 2 diabetes (T2D), and neuropsychiatric disorders. We are pleased to introduce the following Special Issue, “Apolipoproteins and Lipoproteins in Health and Disease 2.0”, which aims to provide a comprehensive overview of the crucial roles of apolipoproteins and lipoproteins across diverse biological processes and disease pathways.

It is widely established that patients with metabolic syndrome (MetS), characterized by elevated triglyceride (TG) levels, decreased levels of HDL-C, and increased levels of small dense LDL particles (sd-LDL), exhibit alterations in lipoprotein structure and function. A study led by Dr. Pintó investigated the impact of the Mediterranean diet (MedDiet) and weight loss on lipoprotein subclasses within the PREDIMED-Plus trial, utilizing advanced lipoprotein testing (ADLT) based on nuclear magnetic resonance spectrometry (NMR) [1]. Specifically, MetS patients were assigned to a group consisting of an energy-reduced MedDiet (er-MedDiet) with physical activity (PA) promotion or a control group. After 12 months, er-MedDiet + PA participants exhibited significant weight loss, decreased triglyceride and LDL-C levels, and increased HDL-C levels. ADLT revealed reductions in sd-LDL-cholesterol and TG content in very-low-density lipoprotein and HDL particles, along with increased LDL particle size. The results of this study suggest that the er-MedDiet + PA diet may attenuate cardiovascular risk compared to the ad libitum MedDiet.

In this context, the results of the study by García-Rodríguez et al. [2] revealed that obese adolescents showed elevated TG concentrations both in fasting and postprandial states, along with increased ceruloplasmin levels during the postprandial period. A higher TG/apo-B48 ratio suggested larger triglyceride-rich lipoprotein (TRL) particle size postprandially, indicating impaired clearance. These differences might stem from variations in the fatty acid composition of postprandial TRL between obese and normal-weight individuals. Specifically, obese subjects showed higher levels of n-6 polyunsaturated fatty acids (PUFAs), such as arachidonic acid, suggesting increased TRL hydrolysis and release of pro-inflammatory adipokines. Conversely, TRL from normal-weight individuals contained higher concentrations of oleic acid (monounsaturated fatty acid) and docosahexaenoic acid (DHA, omega-3 PUFA), potentially exerting anti-inflammatory effects postprandially. These findings highlight the intricate relationship between postprandial lipid metabolism, inflammatory markers, and adipokines in adolescent obesity.

In the study by Peña-de-la-Sancha et al. [3], the authors investigated whether omega-3 PUFAs, eicosapentaenoic acid (EPA) and DHA, could enhance the vascular benefits of HDLs. In a placebo-controlled crossover clinical trial involving hypertriglyceridemic patients, participants received EPA and DHA supplements or a placebo for 5 weeks, followed by a 4-week washout period before crossover. HDLs were isolated and analyzed to determine their fatty acid content. The study results showed that omega-3 PUFA supplementation led to reduced body mass index, waist circumference, and TG and HDL-TG levels, while HDL-C and HDL-phospholipid levels increased. EPA and DHA content in HDLs increased significantly, while omega-6 PUFA levels decreased. The EPA-to-arachidonic acid ratio within HDLs doubled, indicating enhanced anti-inflammatory properties. Despite these changes, the results of in vitro experiments did not show improved endothelial function directly attributed to HDL modification. However, endothelial function improved overall in hypertriglyceridemic patients following omega-3 supplementation. The study authors concluded that EPA and DHA supplementation improves vascular function and modifies HDL composition, potentially enhancing its anti-inflammatory properties and thereby suggesting potential cardiovascular benefits.

In the study by Bonilha et al. [4], the authors investigated the impact of combining evolocumab and empagliflozin versus the use of empagliflozin alone on HDL subspecies in individuals with T2D. Analyzing data from the EXCEED-BHS3 trial, the study authors divided 110 T2D patients into two groups and compared the effects of a 16-week treatment period. Both therapies led to a modest increase in HDL-C levels, with empagliflozin alone and in combination with evolocumab showing similar efficacy. However, the combination therapy resulted in a more pronounced increase in specific HDL subspecies, particularly HDL3b and 3c, compared to the use of empagliflozin alone. Overall, both treatment regimens yielded a modest but significant rise in HDL-C over the 16-week period, with variations observed in the increase in smaller-sized HDL particles between the groups, which could have cardiovascular benefits in T2D.

The p.(Tyr400_Phe402del) mutation in the LDL receptor (LDLR) gene is the predominant cause of familial hypercholesterolemia (FH) in Gran Canaria. In the study by Suarez et al. [5], the authors aimed to determine the mutation’s age and origin and assess its functional impact. Haplotypic analysis of 14 microsatellite loci surrounding the mutation in one homozygous individual and 11 unrelated heterozygous family trios revealed eight distinct mutation carrier haplotypes. These haplotypes were traced back to a common ancestral haplotype, estimated to have originated around 387 years ago following Spanish colonization. The results of functional studies demonstrated that the expressed LDLR was retained in the endoplasmic reticulum, impeding its migration to the cell surface.

As global warming persists, heat stress (HS) in broilers is becoming a significant concern for the poultry breeding industry. The results of numerous studies have shown that HS induces hepatic lipid metabolism disorders in broilers by triggering endoplasmic reticulum (ER) stress. In a recent study by Wang et al. [6], the protective effects of four selenium (Se) sources (sodium selenite, selenium yeast, selenomethionine, and nano-Se) against HS-induced hepatic lipid metabolism disorder and the corresponding selenotranscriptome response in broiler liver were assessed. The study results revealed that dietary supplementation with all Se sources demonstrated similar protective effects. These effects included increased liver Se concentration, enhanced antioxidant capacity, and alleviation of HS-induced ER stress, ultimately mitigating hepatic lipid metabolism disorders in broilers.

Neuropsychiatric disorders (NDs), including schizophrenia and bipolar disorder, are globally prevalent, impacting both the mental and physical well-being of affected individuals. Their multifaceted origins involve genetic, environmental, and stress-related factors. With diverse clinical manifestations, NDs pose challenges for accurate diagnosis. Emerging biomarkers such as apoD offer potential for diagnosis, prognosis, and treatment advancement. In a comprehensive review by del Valle et al. [7], ApoD’s clinical utility as an ND biomarker is explored. The altered expression of apoD in aging and neuropathological conditions, such as schizophrenia and bipolar disorder, suggests its relevance. However, further investigation is warranted to fully assess its diagnostic value. The study’s authors recommend future studies to expand patient cohorts, consider gender influences, and carefully select methodologies for improved insights.

In a noteworthy review by Lee et al. [8], the authors focused on the rhythmic expression of clock genes and lipid-related apolipoprotein genes within the suprachiasmatic nucleus (SCN) of Mus musculus. Circadian rhythm, governing various physiological processes over a 24-h cycle, profoundly influences sleep patterns, body temperature, and hormone levels, with implications for overall health. Disruptions to this rhythm are implicated in numerous diseases, including neurodegenerative conditions. Clock genes within the SCN play a pivotal role in regulating these rhythms, thereby impacting lipid metabolism. Specifically, the review highlights nine apolipoprotein genes, including *apoE*, *apoC1*, and *apoA1*, which exhibit rhythmic expression patterns, potentially under the control of the master clock. Consequently, the authors suggest that understanding these intricate mechanisms holds promise for elucidating the pathophysiology of neurological disorders and may inform future research endeavors, such as employing conditional knockout mice models.

Lastly, Dybiec et al. [9] reviewed the existing knowledge of drugs such as alirocumab and bempedoic acid, which lower LDL-C levels and exhibit promising cardiovascular benefits. Inhibitors of angiopoietin-like protein 3 and apoC3 present novel mechanisms for lowering TG and LDL-C levels, with implications for diseases such as FH. Lomitapide offers an alternative lipid-lowering approach, especially for homozygous FH patients. Moreover, the use of cholesteryl ester transfer protein inhibitors could potentially improve glucose metabolism by enhancing HDL functions. Overall, these new drugs, combined with lifestyle modifications, offer promising avenues for effectively managing dyslipidemia and controlling cholesterol levels.

In conclusion, we believe that the articles and reviews contributed by experts specialized in various diseases will furnish researchers in the apolipoprotein and lipoprotein field with invaluable new data and resources, potentially paving the way for innovative biomarkers or novel therapeutic strategies.

## Abbreviations

ADLT	Advanced lipoprotein testing
Apo	Apolipoprotein
DHA	Docosahexaenoic acid
EPA	Eicosapentaenoic acid
Er-MedDiet	Energy-reduced Mediterranean Diet
HDL	High-density lipoprotein
HDL-C	HDL-cholesterol
HS	Heat stress
CVD	Cardiovascular disease
CKD	Chronic kidney disease
ER	Endoplasmic reticulum
LDL	Low-density lipoprotein
LDL-C	LDL-cholesterol
LDLR	LDL receptor
MedDiet MetS	Mediterranean diet Metabolic syndrome
ND	Neuropsychiatric disorders
NMR	Nuclear magnetic resonance
PA	Physical activity
PUFA	Polyunsaturated fatty acids
SCN	Suprachiasmatic nucleus
Se	Selenium
T2D	Type 2 diabetes
TG	Triglycerides
TRL	Trygliceride-rich lipoproteins
Sd-LDL	Small dense LDL particles
